# Regulation of intestinal flora in patients with chronic atrophic gastritis by modified Chai Shao Liu Jun Zi decoction based on 16S rRNA sequencing

**DOI:** 10.1097/MD.0000000000037053

**Published:** 2024-02-09

**Authors:** Chongyi Xing, Yuna Liu, Shaohua Wang, Jing Zhang, Gang Liu, Na Li, Yan Leng, Dashi Ying, Chunfeng Xu

**Affiliations:** a Changchun University of Chinese Medicine, Changchun, China; b Clinical Laboratory, Beijing Hospital of Integrated Traditional Chinese and Western Medicine, Beijing, China; c Department of Gastroenterology, Beijing Hospital of Integrated Traditional Chinese and Western Medicine, Beijing, China; d Beijing Hospital of Integrated Traditional Chinese and Western Medicine, Beijing, China; e Laboratory of Molecular Pharmacology of Traditional Chinese Medicine, Jilin Ginseng Academy, Changchun University of Traditional Chinese Medicine, Changchun, China; f Affiliated Hospital of Changchun University of Chinese Medicine, Changchun, China; g Jilin Agricultural Science and Technology University, Jilin, China.

**Keywords:** 16S rRNA, Chai Shao Liu Jun Zi decoction, Chinese medicine, chronic atrophic gastritis, intestinal flora

## Abstract

Chai Shao Liu Jun Zi decoction (CSLJZD) is an effective Chinese medicine for the treatment of chronic atrophic gastritis (CAG). However, the effect of CSLJZD on the intestinal flora of patients with CAG remains unclear. We used 16S rRNA gene sequencing to investigate the regulatory effects of CSLJZD on intestinal microflora in patients with CAG. Eight patients with CAG were randomly selected as the model group and 8 healthy medical examiners as the control group; the treatment group comprised patients with CAG after CSLJZD treatment. High-throughput sequencing and bioinformatics analysis of the V3V4 region of the 16S rRNA gene of intestinal bacteria obtained from the intestinal isolates of fecal specimens from all participants were performed separately. A rarefaction curve, species accumulation curve, Chao1 index, and ACE index were calculated to assess the alpha diversity. Principal component analysis (PCA), non-metric multi-dimensional scaling, and the unweighted pair group method with arithmetic mean were used to examine beta diversity. The LEfSe method was used to identify the differentially expressed bacteria. Differential function analysis was performed using PCA based on KEGG function prediction. Rarefaction and species accumulation curves showed that the sequencing data were reasonable. The Chao1 and ACE indices were significantly increased in patients with CAG compared with those in the healthy group. Following CSLJZD and vitacoenzyme treatment, Chao1 and ACE indices decreased. The PCA, non-metric multi-dimensional scaling, and unweighted pair group method with arithmetic mean results showed that the CAG group was distinct from the healthy and treatment groups. The LEfSe results showed that the abundances of the genus *Bilophila*, family Desulfovibrionaceae, order Desulfovibrionales and genus *Faecalibacterium* were significantly higher in the healthy group. The abundance of genus *Klebsiella*, order *Deltaproteobacteria*, genus *Gemmiger*, and other genera was significantly higher in the treatment group. Treatment with CSLJZD had a therapeutic effect on the intestinal flora of patients with CAG.

## 1. Introduction

Chronic atrophic gastritis (CAG) is a common inflammatory disease of the digestive system.^[1]^ The clinical manifestations of the disease are stomach and epigastric pain, fullness, belching, and anorexia. CAG is characterized by chronic inflammatory cell infiltration of the gastric mucosa with reduced intrinsic glands, with or without intestinal epithelial or, atypical hyperplasia. Simultaneously, it is often considered a high risk factor for gastric cancer.^[2,3]^ According to relevant research statistics, the prevalence of gastritis in young age-groups, or even in childhood, is much higher than 50% in developing populations.^[4]^ At the same time, the incidence of CAG in the Chinese normal population is on the rise, with a prevalence of 20%.^[5]^

CAG is a generally asymptomatic condition of great importance because it develops into gastric cancer in many patients. There are 2 types of atrophic gastritis: a gastric body-predominant type in patients with *Helicobacter pylori* (H pylori) infection, and an autoimmune type limited to the gastric body and fundus.^[1]^ The first type is the most common in China, and autoimmune gastritis is rare. Various factors contribute to gastritis, including *H pylori* infection, bile reflux into the stomach, use of non-steroidal anti-inflammatory drugs, dietary imbalances, alcohol and acid exposure, and chronic stress.^[6]^ *H pylori* was also considered a class I carcinogen in a study showing that a proportion of patients with CAG can develop gastric cancer. Therefore, the therapeutic management of the disease urgently requires effective drugs to stop disease progression.

Modern medical measures for CAG mainly include endoscopic follow-up, folic acid supplementation,^[7]^ antioxidants, and eradication of *H pylori*,^[8]^ but the efficacy is hardly satisfactory and the disease effects are difficult to reverse in already atrophied gastric mucosa. Traditional Chinese medicine (TCM) has unique advantages for the treatment of CAG. In China, the use of TCM is becoming increasingly popular for the treatment of diseases. Six Chinese herbs comprise Liu Jun Zi decoction including *Panax ginseng* C. A. Mey (Renshen), *Atractylodes macrocephala* Koidz (Baizhu), *Poria cocos* (Schw.) Wolf (Fuling), Licorice (Gancao), *Citrus reticulata* (Chenpi), and *Arum ternatum* Thunb (Banxia). However, in the present study, the addition of the flavor Chai Shao Liu Jun Zi decoction (CSLJZD) differs from the ordinary Six Jun Zi Tang.

This herbal formula was created to treat CAG with the treatment principle of dredging the liver, strengthening the spleen, and resolving blood stasis by adding flavor to CSLJZD. This is an empirical formula for the treatment of CAG. The formula uses Chai Shao Liu Jun Zi Tang as the base formula for addition and subtraction, and has been effective in the clinical treatment of CAG. In this study, we added Semen Coici, *Fructus aurantii, Cyperus rotundus, Panax pseudoginseng, Curcumae rhizoma* and *Scleromitrion diffusum* bas on this formula, to achieve the efficacy of dredging the liver and strengthening the spleen, invigorating blood circulation and removing blood stasis. The effect of the soup on the intestinal flora of CAG is still unclear, but the study showed that the dysbiosis of intestinal flora is also a causative factor of CAG. Therefore, this study explores the mechanism of the effect of the formula on the treatment of CAG from the perspective of intestinal flora and provides a basis for the clinical application of the Chinese formula.

## 2. Materials and methods

### 2.1. Study design and participants

The study participants were healthy medical examiners and patients with CAG who visited the Beijing Hospital of Integrated Traditional Chinese and Western Medicine between January 2021 and June 2023. All participants were divided into 3 groups: the healthy group, randomly selected medical examiners; the CAG group, patients with CAG who met the inclusion criteria; and the treatment group, patients with herbal prescriptions for CAG. “The Consensus on Chronic Gastritis in China in 2012” published by Members of the Chinese Society of Gastroenterology, was referenced for diagnosing gastritis.^[9]^ The trial was conducted in accordance with the principles of good clinical practice and the Declaration of Helsinki. Informed consent was obtained from all participants at the time of enrollment.

This study was approved by the Beijing Hospital of Integrated Traditional Chinese and Western Medicine. All methods were performed in accordance with the guidelines.

### 2.2. Inclusion criteria and exclusion criteria

Inclusion criteria were as follows: Age 18 to 75 years old; compliance with the Western medical diagnostic criteria and Chinese medical evidence criteria of this disease; Western medical diagnostic criteria: refer to the 2017 Chinese Consensus Opinion on Chronic Gastritis and the 2017 Consensus Opinion on Integrative Diagnosis and Treatment of CAG in Chinese and Western Medicine for the CAG endoscopy and pathology-related diagnosis; Chinese medicine diagnostic criteria Refer to the diagnostic criteria for liver depression and spleen deficiency syndrome in the Guiding Principles for Clinical Research of New Chinese Medicines (Trial Implementation) (2002, China Medical Science and Technology Press).^[10]^ Primary symptoms: fullness or distension of the stomach and epigastrium or ribs; irritability or depression.

Secondary symptoms: poor appetite and dullness; good resting; belching and acidity; tiredness and fatigue; loose stools. Symptoms of the tongue: pale red tongue with greasy moss and teeth marks on the sides; sunken or thin pulse. (Diagnosis of symptoms: The above tongue and pulse are necessary, and 2 of the main symptoms and 2 of the secondary symptoms can be recognized as evidence of Liver Depression and Spleen Deficiency.) No TCM or western medicine for the treatment of this disease within 14 days; endoscopic and pathologic diagnosis results not more than 2 months before enrollment; agreed to participate in this study and signed an informed consent form.

The exclusion criteria were as follows: Those with peptic ulcer, peptic hemorrhage, suspected malignant tendency in pathological diagnosis of gastric mucosa, or those with severe heterotrophic hyperplasia; those whose anxiety or depression self-measurement scale is severe; combination of primary diseases such as heart, brain, liver, kidney or hematopoietic system; those who are allergic to the drugs related to this test; pregnant or lactating women; failure to take the drug on time or failure to comply with medical advice; people taking psychotropic drugs.

### 2.3. Modified CSLJZD and interventions

The modified CSLJZD contains *Bupleurum chinense* DC. (Chaihu)-10g, *Paeonia lactiflora* Pall. (Baishao)-10g, *Codonopsis pilosula* (Dangshen)-12g, *A macrocephala* (Baizhu)-15g, *Pinellia ternata* Thunb (Banxia)-9g, *Citrus reticulate Balanco* (Chenpi)-10g, *Coix lacryma-jobi* (yiyiren)-30g, *Citrus aurantium* L. (Zhiqiao)-10g, *C rotundus* L. (Xiangfu)-10g, *Panax notoginseng* (Sanqi)-3g, *Curcuma phaeocaulis* Val. (Eshu)-9g, *S diffusum* (Baihuasheshecao)-30g, *Corydalis yanhusuo* W. T. Wang (Yuanhu)-15g. The prescription was administered orally 1 hour after meals, twice a day for 4 weeks. The intervention was conducted for 12 weeks in 2-week sessions for a total of 6 sessions. Chinese herbal remedies used for the treatment of CAG are listed in Table 1.

**Table 1:** Ingredients of herbal remedies for chronic atrophic gastritis.

Chinese herbal remedies	Composition of formula	Pharmacological mechanism
Sijunzi decoction	Radix Codonopsis pilosulae, Poria, Rhizoma *Atractylodis macrocephalae*, and Radix glycyrrhizae.	Sijunzi decoction can ameliorate the local gastric inflammation and inflammations in peripheral blood leukocytes and might also reduce the incidence of stomach cancer in chronic gastritis.
Shengyang Yiwei decoction	Astragalus, Codonopsis 30 g, Poria 20 g, 15 g each of white peony root, bark lotus, *Hedyotis diffusa*, and *Atractylodes*, 10 g each of tangerine peel, *Saposhnicovia divaricata, Pinellia, Alisma*, and 5 g licorice.	It regulates the levels of various serum tumor markers in patients and controls the disease.
Liujunzi decoction	Aucklandia lappa, Amomum uillosum, Citrus reticulata, Pinellia ternata, Panax ginseng, Atractylodes macrocephala, Porida cocos, and Glycyrrhiza uralensis.	XSLJZD can improve gastrointestinal motility disorder in functional dyspepsia with spleen deficiency syndrome, which was related to reconstruction of the mitochondrial quality control system by restraining PINK1/Parkin-mediated mitophagy and division.
Huangqi Jianzhong Tang	Radix Astragali (Huangqi), Paeoniae Radix Alba (Baishao), Ramulus Cinnamomi (Guizhi), Rhizoma Zingiberis Recens (Shengjiang), Radix Glycyrrhizae (Gancao), Fructus Jujube (Dazao), and Saccharum Granorum (Yitang).	HQJZ could protect from CAG by altering the mitochondrial function.
Buzhong Yiqi decoction	Huangqi, gancao, dangshen, Danggui, chenpi,shenma, chaihu, baizhu.	BZYQD administration decreased serum levels of the inflammatory factors IL-1β and TNF-α, inhibited phosphorylation of the nuclear transcription factor NF-κB, and down-regulated expression of the inflammatory factors IL-1β and IL-6 in the constipated rat colon.

### 2.4. Sample collection

The clinical information of all the participants is recorded in Table 1. We prepared sterile disposable curved trays, 40 mL stool cups, sterile sampling rods, sealed bags and 2 mL centrifuge tubes that had been sterilized for all participants. The participants were instructed to collect a fresh stool sample (10 g/person) at the hospital, place it in a sterile stool cup, and seal the sample in a foam box with a blue ice pack or refrigerator after collection. As soon as possible (within 24 hours), 2 g of uncontaminated, unexposed stool was sampled in the middle section of stool with a sterile cotton swab, then dispensed into 3 sterilized 2 mL centrifuge tubes and stored at −80ºC.

### 2.5. DNA extraction and 16S rRNA gene sequencing

Microfloral DNA in fecal samples was extracted using a Stool DNA Extraction Kit (TD601-50, Jianshi Bio, China) and a Nano Drop1000 nucleic acid quantitative instrument (Maestro, Nano) was used to detect the DNA content of fecal microflora. DNA integrity and size were checked by 1.0% agarose gel electrophoresis, and the DNA extracts were stored at −80ºC.

Polymerase chain reaction (PCR) amplification of the variable region of the 16S rRNA V4 region was performed according to the PCR procedure instructions and the relevant amplification reaction system and requirements were determined by a pre-experiment. To ensure that the amplified products were not the result of nonspecific amplification, they were analyzed by agarose gel electrophoresis, and the obtained electrophoretic bands were all at approximately 200 bp. The products were sequenced on the MiSeq platform (Illumina Inc., San Diego, CA, USA).

### 2.6. Bacterial community analysis

All data were analyzed using big data analysis techniques. Operational taxonomic units (OTUs) were clustered using QIIME v.2019.10 (https://qiime2.org). Based on the relative OTU abundance table, we assess α-diversity (within-sample diversity; richness using the observed OTUs), β-diversity (diversity of microbial community structure), and comparative analysis at phylum and genus level. Representative sequences from each OTU based on 80% sequence similarity and taxonomic classifications were assigned using the SILVA reference alignment.^[11]^ Based on the above results, the bacterial colony structures in the samples were analyzed for comparative genera and species. Differential function analysis was performed using principal component analysis (PCA) based on KEGG function prediction.

### 2.7. Identification of genomic features using the LEfSe method

Linear discriminant analysis (LDA) effect size (LEfSe) analysis (http://huttenhower.sph.harvard.edu/lefse/) was used to support high-dimensional class comparisons with a focus on comparing species with significant differences.^[12]^ The Kruskal–Wallis rank sum test was first used to determine whether OTU abundance differed between weight groups. LDA was performed (LDA > 0.25, *P* < .05) to estimate the effect size of each differentially abundant trait. The larger the LDA score, the greater the influence of species abundance on the difference.

### 2.8. Statistical analysis

Statistical quantitative analyses and data processing were performed using SPSS 26.0. Normal distribution and homogeneity of variance were tested and the data were expressed as mean ± standard deviation (x̄ ± s). Statistical differences among groups were determined using Student *t* test, and the rank-sum test was used for data not normally distributed. The χ^2^ test (Pearson Chi-square) was used to calculate between-group differences in count data. Correlation analysis between variables was performed using the Pearson linear correlation analysis. Statistical significance was set at *P* < .05 for all analyses.

## 3. Results

### 3.1. Sequencing data preprocessing and quality control

In total, 2214,884 raw sequences were obtained after the 16S rRNA sequencing of the 3 groups of samples. After splicing, filtering and de-embedding the raw data, 1820,546 clean data sequences were obtained, containing 59,292 to 95,501 sequences per sample (Table 2).

**Table 2:** The effective sequence number and sequence length of the sample.

Group	Input	Filtered	Percentage of input passed filter	Denoised	Merged	Percentage of input merged	Non-chimeric	Percentage of input non-chimeric
H1	96906	76325	78.76	75798	72038	74.34	64757	66.82
H2	88638	73195	82.58	72759	70441	79.47	65123	73.47
H3	88945	73465	82.6	72981	69755	78.42	65941	74.14
H4	115104	94295	81.92	93153	86563	75.2	66080	57.41
H5	89446	73960	82.69	73636	72121	80.63	67221	75.15
H6	129444	103570	80.01	102759	95844	74.04	65093	50.29
H7	61153	52277	85.49	51611	48582	79.44	46142	75.45
H8	92712	76779	82.81	76194	72398	78.09	68571	73.96
C1	91602	75460	82.38	74929	71533	78.09	67938	74.17
C2	93169	76987	82.63	76402	72858	78.2	67694	72.66
C3	83632	69303	82.87	69024	67168	80.31	65605	78.44
C4	81331	67286	82.73	67092	66598	81.89	65529	80.57
C5	130577	106281	81.39	105436	98667	75.56	68693	52.61
C6	63747	54303	85.19	53752	51799	81.26	51184	80.29
C7	88641	73972	83.45	73479	70916	80	68274	77.02
C8	86687	70884	81.77	70444	67807	78.22	65886	76
T1	88968	74759	84.03	74416	71969	80.89	69176	77.75
T2	88556	73708	83.23	73171	69736	78.75	66105	74.65
T3	106325	83008	78.07	82281	76687	72.13	65265	61.38
T4	86973	72233	83.05	71573	67690	77.83	64862	74.58
T5	80634	66411	82.36	66210	65343	81.04	64111	79.51
T6	104368	85103	81.54	84143	76908	73.69	68824	65.94
T7	89284	72873	81.62	72392	69404	77.73	65069	72.88
T8	88042	74109	84.17	73709	71919	81.69	65290	74.16

C = CAG group, CAG = chronic atrophic gastritis, H = healthy group, T = treatment group.

### 3.2. Venn diagram

**Figure 1.**
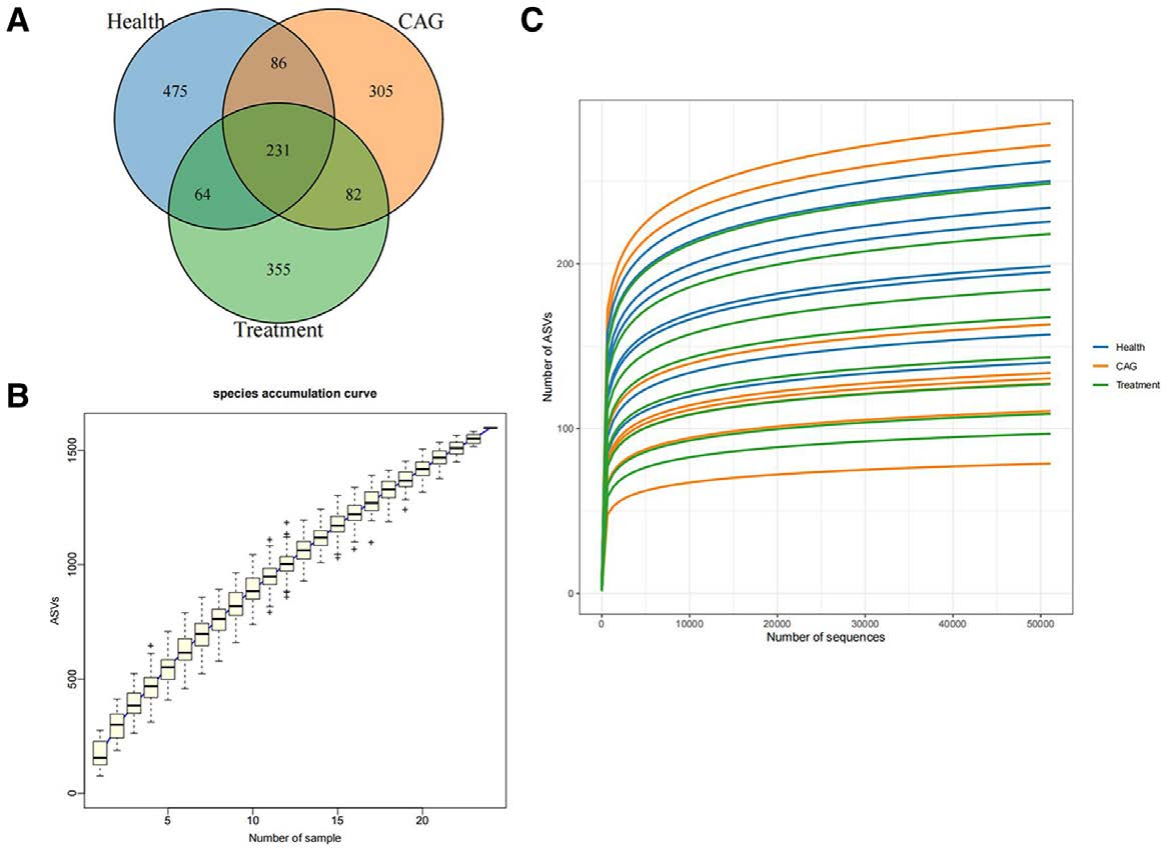
Venn diagram and Alpha diversity indices. (A) Venn diagram of the distribution of the number of OTUs in the sample is plotted, with blue representing health, orange representing CAG, and green representing treatment. (B) Species accumulation curves. (C) Rarefaction curve. CAG = chronic atrophic gastritis, OTUs = operational taxonomic units.

Venn diagrams were used to analyze unique and shared species information in different sample groups at different taxonomic levels (Fig. 1A). We identified 856, 704 and 732 species in the healthy, CAG, and treatment groups, respectively, of which 231 species were the same and the others were not common to all groups; 475 were unique to the healthy group, 305 were unique to the CAG group and 355 were unique to the treatment group.

### 3.3. Alpha diversity

#### 3.3.1. Rarefaction curve.

By comparing the dilution curves of different samples, the differences in species richness between samples were visualized, and used to assess whether the sequencing volume of a sample was reasonable. When the curve flattens out as the number of extracted sequences increases in the dilutability graph, it indicates that the amount of sequencing data in the sample is reasonable.

Species accumulation curves can be used to determine whether a sample size is sufficient. As shown in Figure 1C, the dilution curve gradually flattened, indicating that the sequencing data in this study were reasonable.

#### 3.3.2. Species accumulation curves.

Species accumulation curves are used to describe the increase in species with increasing sample size, and are widely used to determine the adequacy of sample size and estimate species richness. If the curve shows a sharp increase, it indicates that a large number of new species will be found as the sampling volume increases, that is, the sampling volume is insufficient. If the curve tends to flatten, it indicates that the species in this environment will not increase significantly with the increase in sample size, which indicates that the sampling is sufficient. As the sample size increased, the box plot flattened, indicating that the sample size was sufficient (Fig. 1B).

Chao1 is used to estimate the total number of species in the sample, with larger values representing more species. ACE was used to estimate the total number of species in the sample, with larger values representing more species, using a different algorithm than Chao1. Shannon index was used to estimate the diversity indices of the microorganisms in the sample. The Simpson diversity index is a commonly used index that reflects the alpha diversity. Higher Shannon index values indicated higher community diversity.

**Figure 2.**
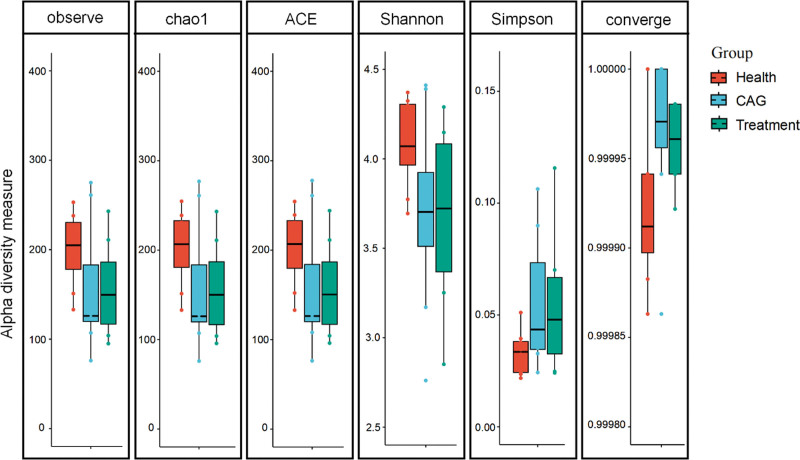
Alpha diversity indices from different groups. The figure shows the 6 diversity indices for each group of samples, with the horizontal coordinates indicating the different groups and the vertical coordinates indicating the community diversity index values for that group of samples, with different groups distinguished using different colors. Orange representing health, blue representing CAG, and green representing treatment. CAG = chronic atrophic gastritis.

The 16S rRNA V3V4 region in the participant fecal bacteria was amplified by PCR and high-throughput sequenced, clustered into OTUs under the condition of preserving 97% similarity, and the sample abundance information in each OTU was counted. The library coverage (coverage) indices of the samples from both groups were >0.99, indicating a relatively low probability of unmeasured sequences in the samples. There were no significant differences between the ACE and Chao indices of the 3 groups (Fig. 2), and the abundance of the 3 groups of colonies was similar. The Chao1 and ACE indices were significantly higher in the CAG group, suggesting a higher intestinal flora diversity in patients with CAG.

### 3.4. Beta diversity

PCA can be used to show between-sample similarities or differences in community composition. Samples with high community structure similarity cluster together, whereas samples with large differences separate.

**Figure 3.**
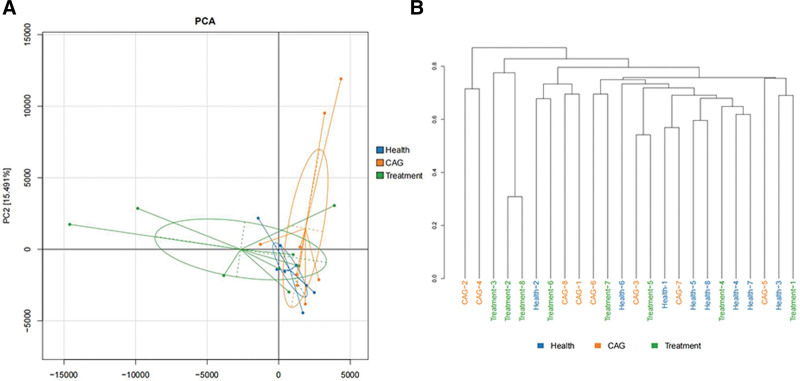
Beta diversity. (A) PCA score plot. (B) UPGMA result. The results showed that the community composition of the treated group differed significantly from that of the CAG group, with a total variation of 15.693, indicating the effect of modified Chai Shao Liu Jun Zi decoction components on the composition of intestinal microorganisms. The NMDS results also showed a separation between the CAG and treatment groups. Blue representing health, orange representing CAG, and green representing treatment. CAG = chronic atrophic gastritis, NMDS = non-metric multi-dimensional scaling, PCA = principal component analysis, UPGMA = unweighted pair group method with arithmetic mean.

PCA is based on the OTU/ASV abundance table and uses variance decomposition to reflect the differences between samples on a 2-dimensional coordinate plot, with the axes representing the 2 characteristic roots that can explain the variance to the greatest extent. The more similar the sample composition, the more aggregated it is in the PCA plot, whereas samples from different groups may exhibit a scattered distribution (Fig. 3A).

#### 3.4.1. UPGMA (unweighted pair group method with arithmetic mean).

Two similar samples cluster preferentially. In addition, the UPGMA was used to compare similarities between different samples. The results showed that most intestinal microorganisms clustered in their own groups, which is consistent with our PCA and non-metric multi-dimensional scaling results (Fig. 3B).

### 3.5. Analysis of horizontal community structures across groups

**Figure 4.**
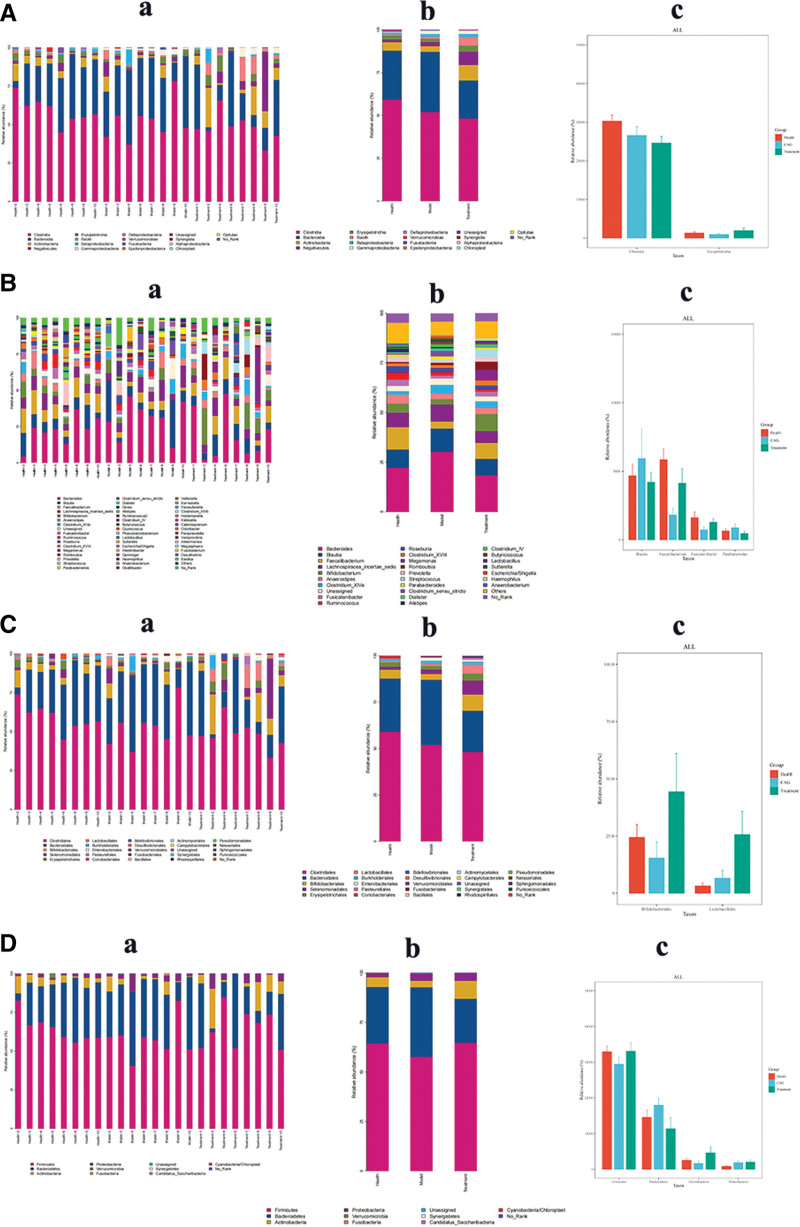
Composition of the intestinal flora community of each group of samples plotted. (A): (a) Histogram of relative abundance of species based on class level. (b) Histogram of relative abundance of species based on class level. (c) Differences in gut flora abundance at the class level. (B): (a) Histogram of relative abundance of species based on genus level. (b) Histogram of relative abundance of species based on genus level. (c) Differences in gut flora abundance at the genus level. (C): (a) Histogram of relative abundance of species based on order level. (b) Histogram of relative abundance of species based on order level. (c) Differences in gut flora abundance at the order level. (D): (a) Histogram of relative abundance of species based on phylum level. (b) Histogram of relative abundance of species based on phylum level. (c) Differences in gut flora abundance at the phylum level. Orange representing health, blue representing CAG, and green representing treatment. CAG = chronic atrophic gastritis.

At the phylum level (Fig. 4D), the CAG group showed a decrease in the abundance of Firmicutes and Actinobacteria, and an increase in the abundance of Bacteroidetes and Proteobacteria compared with the healthy group, and a decrease in the abundance of Firmicutes, Actinobacteria, and Proteobacteria after the CSLJZD treatment. The abundances of Firmicutes, Actinobacteria, and Proteobacteria abundance increased, while Bacteroidetes abundance decreased after CSLJZD treatment.

The analysis was performed at the class and order levels (Fig. 4A and C). At the order level, the abundance of Clostridia and Erysipelotrichia decreased in the CAG group compared with that in the healthy group, and the abundance of Clostridia decreased further after CSLJZD treatment, while the abundance of Erysipelotrichia increased. The same trend was observed at the order and family levels, which may be related to the anti-inflammatory and anti-infectious properties of CSLJZD.

At the genus level (Fig. 4B), the abundance of *Faecalibacterium* and *Fusicatenibacter* decreased compared with the healthy group, and the abundance of *Bacteroides* and *Blautia* increased compared with the healthy group. After treatment with CSLJZD, the abundance of *Erysipelotrichia, Bacteroides*, and *Blautia* decreased, while *Faecalibacterium* and, *Fusicatenibacter* abundances increased.

### 3.6. LEfSe analysis

**Figure 5.**
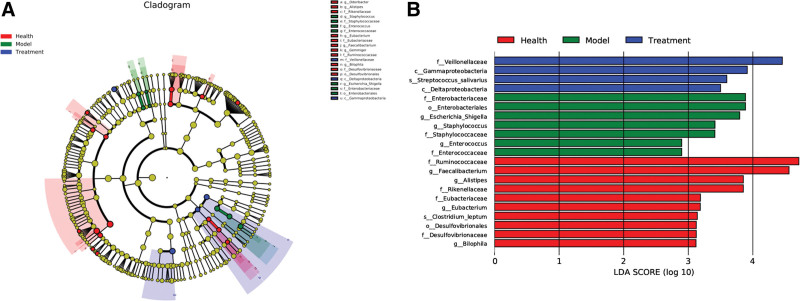
Microbial signatures of gut microflora in in 3 groups. (A) The species that do not differ significantly with respect to abundance are colored yellow. The size of the nodes is consistent with the relative abundance of the species. From inside to outside, the circles represent phylum, class, order, family and genus. Orange representing health, blue representing CAG, and green representing treatment. (B) linear discriminant analysis. CAG = chronic atrophic gastritis.

Potential biomarkers were defined using LEfSe. We used the LEfSe method to identify specific bacterial phylotypes that were differentially altered between the healthy and treatment groups. Cladograms were obtained using the LEfSe method (LDA > 2.0). LEfSe analysis revealed that the following groups had significantly different information (*P* < .05), mainly distributed at the phylum, order, order, family and genus levels. The healthy group included members of the phyla Firmicutes, Clostridia, Clostridiales, Ruminococcaceae, Desulfovibrionaceae, Desulfovibriones, Bilophila, and *Faecalibacterium* (Fig. 5A and B). Their relative abundance was higher than that in the other groups. The CAG group included Gammaproteobacteria, Enterobacteriaceae, Enterobacteriales, *Escherichia coli*, and *Escherichia shigella*. Their relative abundance was higher than that in the other groups. The treatment group included Actinobacteria, Actinobacteria, *Bifidobacteriales, Bifidobacteriaceae, Bifidobacterium, Lactobacillales, Bacilli, Deltaproteobacteria Klebsiella, Blautia, Gemmiger, Clostridium, Ruminococcaceae* and *Faecalibacterium*. Their relative abundance was higher than that in the other groups.

The outer ring of the strips represents the taxonomic affiliation of the OTUs at the phylum level, and other levels of annotation information are shown in the legend to the right. The distribution bar chart of LDA values shows species with LDA scores greater than the set value and species with significantly different abundances in different groups.

### 3.7. PICRUSt2

**Figure 6.**
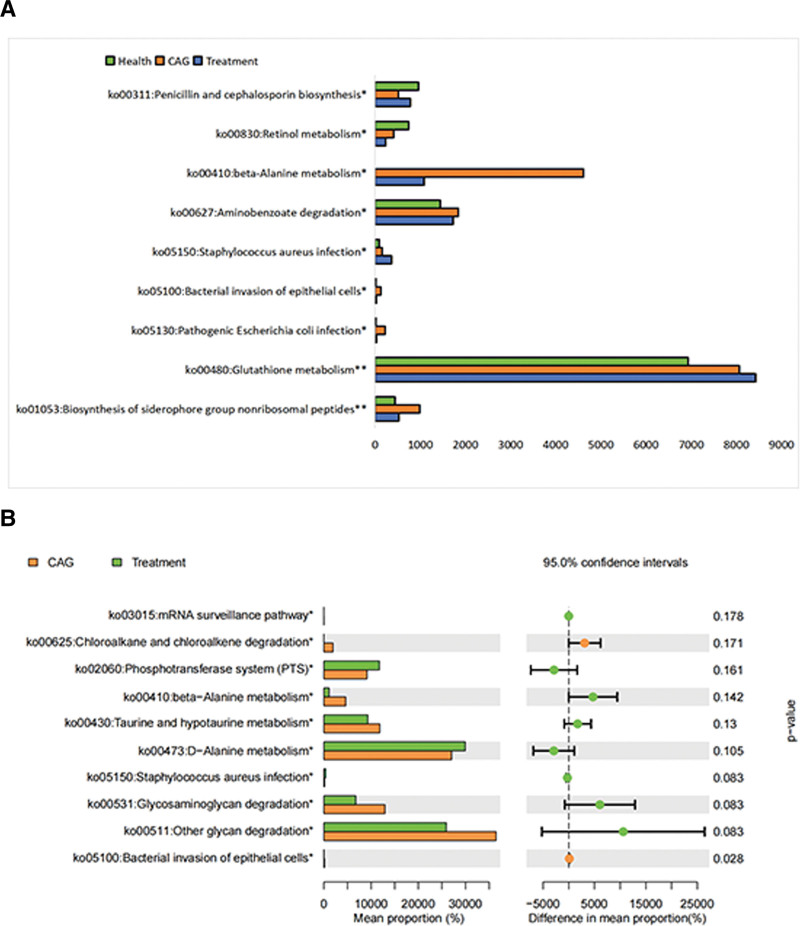
Differences in the relative abundance of function (KEGG) between 2 subgroups of samples were compared using Wilcoxon analysis. (A) Health, CAG and treatment group. (B) CAG and treatment group. CAG = chronic atrophic gastritis.

Differences in the relative abundance of function (KEGG) between the 2 sample subgroups were compared using (Fig. 6A and B) Wilcoxon analysis. The pathways that differed significantly among the 3 groups were penicillin and cephalosporin biosynthesis, retinol metabolism, beta-alanine metabolism, aminobenzoate degradation, *Staphylococcus aureus* infection, bacterial invasion of epithelial cells, pathogenic *E coli* infection, glutathione metabolism, and biosynthesis of siderophore group nonribosomal peptides. The pathways that differed significantly between the CAG and treatment groups were the mRNA surveillance pathway, chloroalkane and chloroalkene degradation, phosphotransferase system, beta-alanine metabolism, taurine and hypotaurine metabolism, d-alanine metabolism, *S aureus* infection, and glycosaminoglycan. According to the above results, the KEGG differential pathways between the CAG and treatment groups were mainly enriched in amino acid metabolic pathways.

## 4. Discussion

Chronic gastritis with erosions belongs to the categories of “epigastralgia,” “stomach heaviness,” and “gastric upset” in TCM. In TCM theory, a syndrome pattern plays an important role in evaluating pathogenesis.^[13]^ CAG has obvious lesions in the stomach, which are closely related to the liver and spleen. Emotional disorders are one of the causes of this disease. In the process of observing clinical patients, CAG is often accompanied by adverse emotions, and after long-term follow-up, it can be concluded that emotional disorders and the occurrence and development of CAG may influence each other. If the patient mood is not smooth, the liver is depressed and qi is stagnant, and the spleen and earth are violated; the spleen loses its health, the stomach loses its harmony and descent, and pathological products such as phlegm, dampness, turbidity, and stasis are lost over time. The qi and blood in the gastric lobe lose harmony and the gastric membrane loses nourishment, thus triggering or aggravating the disease, and the disease recurs; the bad mood disorder will become increasingly serious, and it can often cause the disease in a cycle. The TCM syndrome is also related to *H pylori* infection and gastric mucosal atrophy, and spleen-stomach dampness-heat syndrome is more prevalent in *H pylori* infection.^[14]^ Therefore, the main treatment is to dredge the liver, strengthen the spleen, remove blood stasis, and treat CAG with the addition of CSLJZD.

Modern pharmacological studies^[5]^ have shown that the basic formula Chai Shao Liu Jun Zi Tang, enhances the dynamics of the gastrointestinal tract, contracts the gastric sinus, and accelerates gastric emptying. The formula used in this study-CSLJZD,^[15]^ was effective in clearing the liver and relieving depression, strengthening the spleen and dispelling dampness, harmonizing the stomach and lowering rebelliousness, resolving blood stasis and clearing the ligaments, unblocking qi flow, and pushing out the new, thus alleviating both the symptoms and root cause. White peony relieves smooth muscle spasms and regulates immunity. Radix Bupleuri and white peony can effectively relieve stomach pain and stomach spasms; *A macrocephala* promotes gastrointestinal peristalsis, protects the gastric mucosa and improves immunity. Radix Codonopsis has antioxidant, anti-stress, anti-tumor, and immune-regulatory functions. Radix Aromaticus has a wide range of pharmacological effects, including anti-inflammatory, antibacterial, antioxidant, neuroprotective and anticancer. *Curcuma longa* has anti-inflammatory and analgesic, anti-tumor and anti-depressant effects, enhances gastrointestinal function, improves gastric local blood circulation, enables gastric mucosa reconstruction, inhibits intestinal chemotaxis and heterotypic hyperplasia, and prevents early carcinogenesis. *S diffusum* has anti-inflammatory, antioxidant, anti-multiple tumor and enhanced body immunity effects. Although this formula has achieved good results in the treatment of CAG, its effect on the regulation of intestinal flora is unclear.

A growing body of data suggests that the gut microbiota plays a key role in herbal therapy through complex interactions with herbal components.^[16]^ Herbal preparations can significantly improve the composition of the intestinal flora and its dysfunction, while the intestinal flora may mediate the metabolism of a variety of chemicals in herbal medicines.^[16]^ The 16S rRNA sequencing approach was used to investigate whether the gut microbiota plays a role in the treatment of CAG by CSLJZD, and the relationship between the gut microbiota and differential metabolites. Our current findings suggest that CSLJZD has a significant effect on changes in the intestinal flora, at the phylum and order levels as well as endogenous metabolites and their interactions. These results suggest that the intestinal flora plays an important role in the treatment of CAG with CSLJZD.

The Venn diagram shows that 231 species were identical among the 3 groups. These species may play an important role in the stability and function of the intestinal microecological environment of CAG patients. The CAG group had 305 species, which may have a strong relationship with the onset and development of CAG and is our next research target. Sparsity and species accumulation curves were generated, proving the reliability of the sequencing data. The Chao1 and ACE indices were significantly higher in the CAG group, suggesting that patients with CAG have a more diverse intestinal flora. Previous reports have shown that changes in the microbial composition and diversity are critical for promoting inflammation, proliferation, and tumor progression.^[17]^ Furthermore, it has been shown that abnormal cell proliferation and apoptotic mechanisms play an important role in the malignant transformation of the gastric mucosa.^[18]^ Therefore, we hypothesized that changes in the intestinal flora diversity in patients with CAG may be an important factor in the development of CAG. After treatment with CSLJZD, the Chao1 and ACE indices were reduced, but not significantly, compared with the control group, indicating that CSLJZD could effectively regulate the diversity of intestinal flora in CAG.

According to the analysis of horizontal community structures across groups, the abundance of Clostridia was further reduced and the abundance of Erysipelotrichia increased after JWCST treatment. *Lactobacillus* and *Bifidobacterium* are important components of the human intestinal flora that can improve epithelial and mucosal barrier functions, regulate the composition of the intestinal microbiota and inhibit the proliferation of *H pylori*. They also exert anti-inflammatory effects.^[19]^ Meanwhile, *Bifidobacterium bifidum* and *Lactobacillus lactis* can produce butyrate, acetate and propionate which are the main short-chain fatty acids, and these short-chain fatty acids can inhibit inflammation and cancer in the gastrointestinal tract.^[20]^ In addition, several in vitro and in vivo studies have shown that *Lactobacillus* and *Bifidobacterium* exert some anti-oxidative stress effects.^[21]^ An imbalance in oxidative stress can result in the abnormal activation of the inflammatory response, leading to epithelial-mesenchymal transformation and, ultimately, a decrease in gastric mucosal epithelial cell production, which can lead to the development of CAG.^[22]^ In the present study, the abundance of beneficial bacteria significantly increased after treatment, and the same trend was observed at the subsequent family level, indicating that CSLJZD enhanced the abundance of beneficial bacteria, regulated the structure of the microbial community, played a protective role in the gastrointestinal mucosa, and reduced the inflammatory response to a certain extent, thus effectively inhibiting the occurrence of inflammation-cancer transformation. Studies have shown that *Faecalibacterium* spp. have anti-inflammatory, anxiety-relieving, and depression-relieving effects, that they are highly expressed in gastritis patients with eradication of *H pylori*,^[23]^ that the abundance of *Faecalibacterium* spp. is reduced in patients with liver-depressant and spleen-deficient type of CAG, and that CAG leads to the destruction of beneficial bacteria in the intestinal tract, which makes the microbiota harmful and weakens its protective function. The increase in abundance after treatment suggests that CSLJZD may increase the abundance of *Faecalibacterium* spp., thereby contributing to the treatment of CAG. In addition, studies have shown that the abundance of *Fusicatenibacter* in patients with severe depression and generalized anxiety is significantly lower than that in healthy people,^[24]^ and in patients with CAG, the abundance of *Fusicatenibacter* is lower than that in healthy individuals, and after treatment, the abundance of this bacterial group is regional in the healthy group, suggesting that CSLJZD can improve the state of CAG with anxiety and depression. Modern medicine points out that affective factors can induce CAG by mediating gastric mucosal damage through the neuroendocrine system,^[25]^ and at the same time, the discomfort of some clinical patients is aggravated by mood fluctuations, and an increasing number of studies have shown that the gut microbiota is also crucial for mood and social cognition. The changes in *Faecalibacterium* and, *Fusicatenibacter* observed in the present study provide an argument for this view. Previous studies have shown that *Anaplasma* spp. and *Brautella* spp. are closely related to CAG spleen and stomach damp-heat syndrome, that *Brautella* spp. can activate inflammatory cytokines,^[26]^ which further cause inflammatory responses, and that the abundance of these 2 genera decreased after treatment, which is in line with the results of the present study, suggesting that CSLJZD has an anti-inflammatory effect.

The results of PCA, non-metric multi-dimensional scaling, and UPGMA indicated that the microbial composition of the intestinal flora of CAG patients changed, and that CSLJZD treatment had a therapeutic effect on the intestinal flora community structure in patients with CAG. The KEGG database was used as the basis for functional prediction using the PICRUSt software. In the comparison of glutathione metabolism, the expression of this bioprocess was higher in the CAG group and lower in the treatment group. Penicillin and cephalosporin biosynthesis was significantly decreased in the CAG group and elevated in the treated group, which also suggests that CSLJZD has some anti-inflammatory effect.

Amino acids and their derivatives can act as neurotransmitters or neuromodulators that participate in or regulate neurotransmission processes.^[27,28]^ Wilcoxon analysis revealed that KEGG differential pathways between the CAG and treatment groups were mainly enriched in amino acid metabolism pathways, such as beta-alanine metabolism, taurine and hypotaurine metabolism, and d-alanine metabolism. This finding also suggests that CSLJZD can be involved in regulating the complex connection between endogenous metabolites, the central nervous system and the gastrointestinal tract, which lays an experimental foundation for the subsequent elucidation of the biological mechanism of CSLJZD in the treatment of CAG through liver-sparing, spleen-strengthening and stasis-removing at the metabolic level. During the onset of CAG, patients experience loose stools, anxiety, and depression symptoms, and more and more studies have also shown that there is a close relationship between intestinal flora and brain gut peptides and CAG.^[29]^ The intestinal flora also plays a non-negligible role in brain-gut interactions, and, by participating in the synthesis and release of various hormones, cytokines, and neurotransmitters, directly or indirectly modulates the brain-gut axis, which then affects the brain. CSLJZD can regulate intestinal micro-ecology and help normalize intestinal flora while benefiting qi, tonifying the spleen and relieving liver and depression, thus affecting gastrointestinal function and recovery of mental and psychological states through the brain-intestinal axis.^[30]^

In summary, the results of this study showed that CSLJZD can regulate the intestinal microecological balance in patients with liver-depleted and spleen-deficient CAG, and the diversity of the intestinal flora in patients with liver-depleted and spleen-deficient CAG was improved after treatment with CSLJZD. The structure of the species composition tended to be similar to that of healthy individuals and the abundance of beneficial bacteria was up-regulated, suggesting that regulating the intestinal flora may be a link in the mechanism of the pharmacological efficacy of CSLJZD in the treatment of liver-depleted and spleen-deficient CAG.

## Acknowledgments

We thank the clinicians in this group for their perseverance and dedication, and the editors for their comments on this article.

## Author contributions

**Conceptualization:** Shaohua Wang.

**Data curation:** Yuna Liu.

**Funding acquisition:** Chunfeng Xu.

**Investigation:** Jing Zhang.

**Methodology:** Gang Liu.

**Software:** Na Li, Dashi Ying.

**Validation:** Yan Leng.

**Writing – original draft:** Chongyi Xing.

**Writing – review & editing:** Chunfeng Xu.
